# Massive Pulmonary Embolism in Early Pregnancy Presenting as Syncope and Seizure: A Diagnostic Pitfall

**DOI:** 10.7759/cureus.103237

**Published:** 2026-02-08

**Authors:** Salama Alharmoodi, Humaid Sadiq, Zayed Alhammadi, Mohd Reidwan Dar, Nagaraj V Kumar

**Affiliations:** 1 Emergency Medicine, Sheikh Tahnoon bin Mohammed Medical City, Al Ain, ARE; 2 Emergency Medicine, Tawam Hospital, Al Ain, ARE; 3 Critical Care, Tawam Hospital, Al Ain, ARE

**Keywords:** cardiopulmonary arrest, emergency medicine, generalized tonic clonic seizure, intensive care unit, massive pulmonary embolism, mechanical thrombectomy, pregnancy, right atrial thrombus, saddle pulmonary embolism, syncope

## Abstract

Pulmonary embolism (PE) is a life-threatening condition with highly variable presentations, and atypical manifestations such as syncope or seizure can obscure timely diagnoses, particularly in young and otherwise healthy individuals. This case report focuses on a 26-year-old female in early pregnancy who presented with a transient loss of consciousness and seizure-like activity and was ultimately found to have a massive saddle PE. The case emphasizes the diagnostic challenges and the critical role of rapid, multidisciplinary intervention in such high-risk scenarios. The patient was previously healthy and reported mild lower abdominal discomfort. On presentation, she was hypotensive and tachycardic and had a superficial forearm burn sustained during the event. Laboratory evaluation revealed hypokalemia, elevated lactate, raised creatine kinase, and troponin. Positive β-hCG prompted a pelvic ultrasound, suggesting a possible early gestational sac, with gynecology attributing the findings to an implantation bleed. Neurological and cardiac evaluations excluded seizure disorder and myocardial ischemia. Despite fluid resuscitation, the patient remained hemodynamically unstable. An electrocardiogram (ECG) demonstrated an S1Q3T3 pattern, and bedside echocardiography revealed right ventricular dilation. Computed tomography (CT) pulmonary angiography confirmed extensive bilateral pulmonary emboli, including a saddle embolus. She underwent mechanical thrombectomy complicated by cardiac arrest, requiring cardiopulmonary resuscitation (CPR) and intravenous (IV) alteplase administration, followed by intensive care unit (ICU) care. A right atrial thrombus was noted and managed with anticoagulation. Gynecological assessment revealed a missed abortion. The patient stabilized with supportive care, anticoagulation, and monitoring and was successfully extubated and discharged after 10 days on rivaroxaban, with follow-up arranged with cardiology, pulmonology, and internal medicine. This case underscores the importance of maintaining a high index of suspicion for PE in atypical presentations, particularly in pregnancy, and highlights the critical role of prompt imaging, multidisciplinary collaboration, and timely intervention in improving outcomes for high-risk patients.

## Introduction

Pulmonary embolism (PE) is a potentially life-threatening cardiovascular emergency resulting from the obstruction of the pulmonary arteries, most commonly by thrombus originating from the deep veins of the lower extremities [1,2]. Clinical presentation is highly variable, ranging from mild dyspnea or pleuritic chest pain to sudden cardiovascular collapse [3]. Because of this heterogeneity, diagnosis can often be delayed or missed, particularly in young or otherwise healthy individuals without identifiable risk factors [4].

The incidence of PE is estimated at approximately 120 cases per 100,000 individuals annually, with maternal mortality significantly reduced by early recognition; prompt initiation of anticoagulation and supportive care significantly improves outcomes [1,2]. However, atypical presentations, including syncope or seizure, can obscure the underlying etiology and pose significant diagnostic challenges in the emergency department (ED) [4].

Pregnancy further complicates diagnosis and management due to physiological hypercoagulability and concerns regarding fetal radiation exposure. Maintaining a high index of suspicion, use of validated pregnancy-adapted diagnostic strategies, and early multidisciplinary evaluation are essential to optimize outcomes [5-8].

This case report describes a young, previously healthy female in early pregnancy who presented with transient loss of consciousness and seizure-like activity and was ultimately diagnosed with massive saddle PE, highlighting the importance of recognizing atypical PE presentations and the need for rapid, coordinated intervention in high-risk patients.

## Case presentation

Initial presentation

A 26-year-old previously healthy female presented to the ED after a sudden syncopal episode while walking to work. Upon emergency medical services (EMS) arrival, she developed a brief generalized tonic-clonic seizure with frothy oral secretions that resolved spontaneously. She reported mild lower abdominal discomfort and had sustained a superficial friction burn on the left forearm during the episode. She denied chest pain, palpitations, dyspnea, cough, hemoptysis, or recent long-distance travel. She had no history of oral contraceptive use, smoking, or prior thromboembolic disease, and her family history was unremarkable. The remainder of her review of systems was unremarkable.

Emergency department evaluation

On arrival, the patient was alert and oriented with a Glasgow Coma Scale (GCS) of 15 [9]. She was diaphoretic, tachycardic (140 beats/minute), mildly hypotensive (blood pressure 96/63 mmHg), and tachypneic (respiratory rate 35 breaths/min) on 5 liters of supplemental oxygen, maintaining an oxygen saturation (SpO₂) of 98%. An abdominal exam revealed mild lower abdominal tenderness. Chest and cardiovascular examinations were otherwise unremarkable, and there were no signs of deep venous thrombosis (DVT) noted on the lower limbs.

Initial laboratory investigations revealed hypokalemia (2.8 mmol/L), elevated lactate (4.7 mmol/L), raised creatine kinase (551 U/L) and troponin-I (715 ng/L), normal hemoglobin (140 g/L), and a positive β-hCG level of 975 IU/L (Table 1).

**Table 1:** Initial blood investigation results. Beta-HCG: beta-human chorionic gonadotropin

Test	Result	Reference range	Unit
pH	7.44	7.35–7.45	
pCO2	30.6	35.0–45.0	mmHg
pO2	64.6	25.0–40.0	mmHg
HCO3	21	22–26	mmol/L
Base excess (BE)	-2.2	-2.0–2.0	mmol/L
Total hemoglobin (THB)	140	120–170	g/L
Carboxyhemoglobin (COHB)	0.9	0.0–2.0	%
Potassium (K)	2.8	3.4–5.1	mmol/L
Chloride (Cl)	104	98–107	mmol/L
Sodium (Na)	138	136–145	mmol/L
Glucose (Glu)	14.8	3.9–6.0	mmol/L
Lactate (Lac)	4.7	0.5–2.2	mmol/L
Total creatine kinase (CK)	551	29–168	IU/L
Troponin-I	715.0	≤15.60	ng/L
Beta hCG quant	975.1	≤5.0	milli IU/ml

Toxicology screening and other laboratory parameters were unremarkable. Focused abdominal sonography (FAST) at the bedside was unremarkable.

Multidisciplinary consultations were obtained for further evaluation. Pelvic ultrasound revealed a small (0.7 cm), well-defined endometrial cystic lesion with surrounding decidual thickening, suggesting a possible early gestational sac, which was considered consistent with an implantation bleed by gynecology. Cardiology attributed the elevated troponin to skeletal muscle leak secondary to raised CK, with no cardiac intervention indicated. Neurology found no evidence of cerebral venous thrombosis, and given the brief episode, rapid recovery, and absence of postictal features, a seizure was considered unlikely.

Initial resuscitation and hemodynamic monitoring

The patient received two litres of intravenous (IV) fluids without significant clinical improvement. Repeat venous blood gas (VBG) showed worsening metabolic acidosis with elevated lactate and stable hemoglobin, suggesting ongoing tissue hypoperfusion despite adequate fluid resuscitation (Table 2). Tachycardia persisted (104 bpm), and systolic blood pressure (SBP) remained low (97 mmHg).

**Table 2:** Second venous blood gas results.

Blood gas venous	Result	Reference range	Unit
pH	7.24	7.35–7.45	
pCO2	55.6	35.0–45.0	mmHg
pO2	21.3	25.0–40.0	mmHg
HCO3	24	22–26	mmol/L
Base Excess (BE)	-4.2	-2.0–2.0	mmol/L
Total Hemoglobin (THB)	133	120–170	g/L
Carboxyhemoglobin (COHB)	0.7	0.0–2.0	%
Potassium (K)	3.4	3.4–5.1	mmol/L
Chloride (Cl)	106	98–107	mmol/L
Sodium (Na)	142	136–145	mmol/L
Glucose (Glu)	7.9	3.9–6.0	mmol/L
Lactate (Lac)	5.9	0.5–2.2	mmol/L

A third litre of IV fluid improved acid-base status and lactate, indicating resolving hypoperfusion (Table 3). IV potassium chloride (20 mmol) corrected the hypokalemia. During Foley catheterization, she had a brief generalized tonic-clonic seizure, treated with 1 mg IV lorazepam. Neurology recommended magnetic resonance imaging (MRI) and magnetic resonance venography (MRV), electrolyte correction, and initiation of lamotrigine 25 mg daily. MRI/MRV was unremarkable.

**Table 3:** Third venous blood gas results.

Blood gas venous	Result	Reference range	Unit
pH	7.34	7.35–7.45	
pCO2	42.8	35.0–45.0	mmHg
pO2	19.3	25.0–40.0	mmHg
HCO3	23	22–26	mmol/L
Base excess (BE)	-2.9	-2.0–2.0	mmol/L
Total hemoglobin (THB)	120	120–170	g/L
Carboxyhemoglobin (COHB)	0.9	0.0–2.0	%
Potassium (K)	3.4	3.4–5.1	mmol/L
Chloride (Cl)	110	98–107	mmol/L
Sodium (Na)	142	136–145	mmol/L
Glucose (Glu)	6.7	3.9– 6.0	mmol/L
Lactate (Lac)	3.4	0.5–2.2	mmol/L

**Figure 1:**
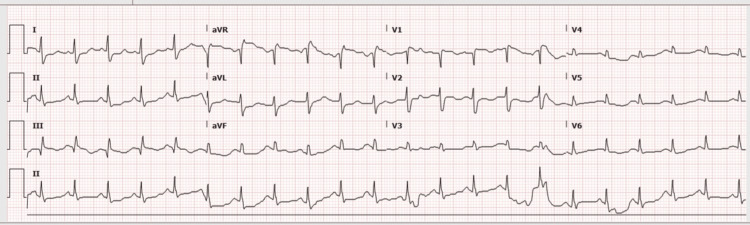
Electrocardiogram (ECG) demonstrating sinus tachycardia and S1Q3T3 pattern.

The patient remained hemodynamically unstable, with persistent sinus tachycardia and hypoxia. ECG showed sinus tachycardia (HR 130 beats per minute) with an S1Q3T3 pattern (Figure 1), and bedside transthoracic echocardiogram (TTE) revealed right ventricular dilation with preserved left ventricular function, raising concern for acute PE.

**Figure 2:**
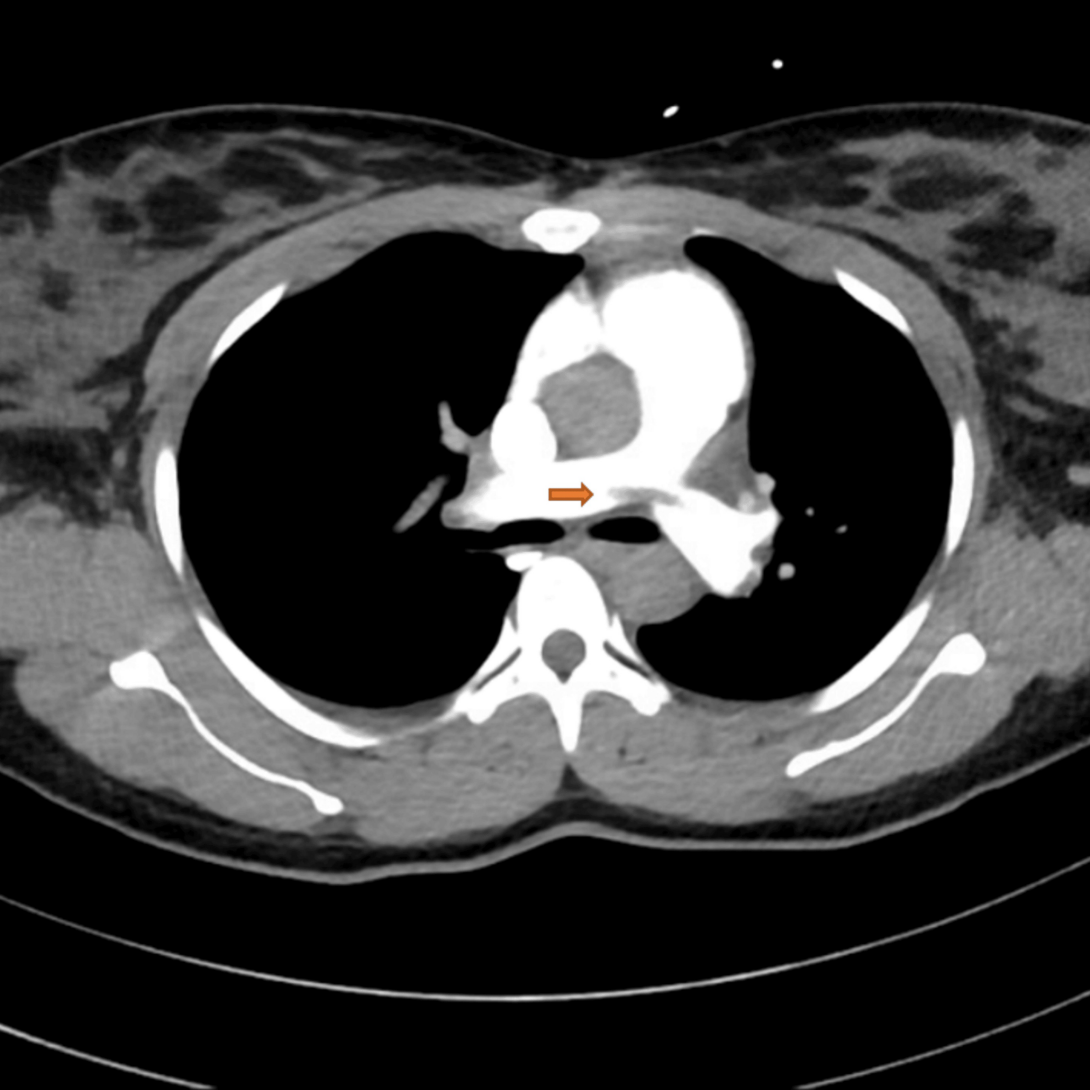
Computed tomography pulmonary angiography (CTPA) showing saddle embolus at the pulmonary trunk bifurcation.

**Figure 3:**
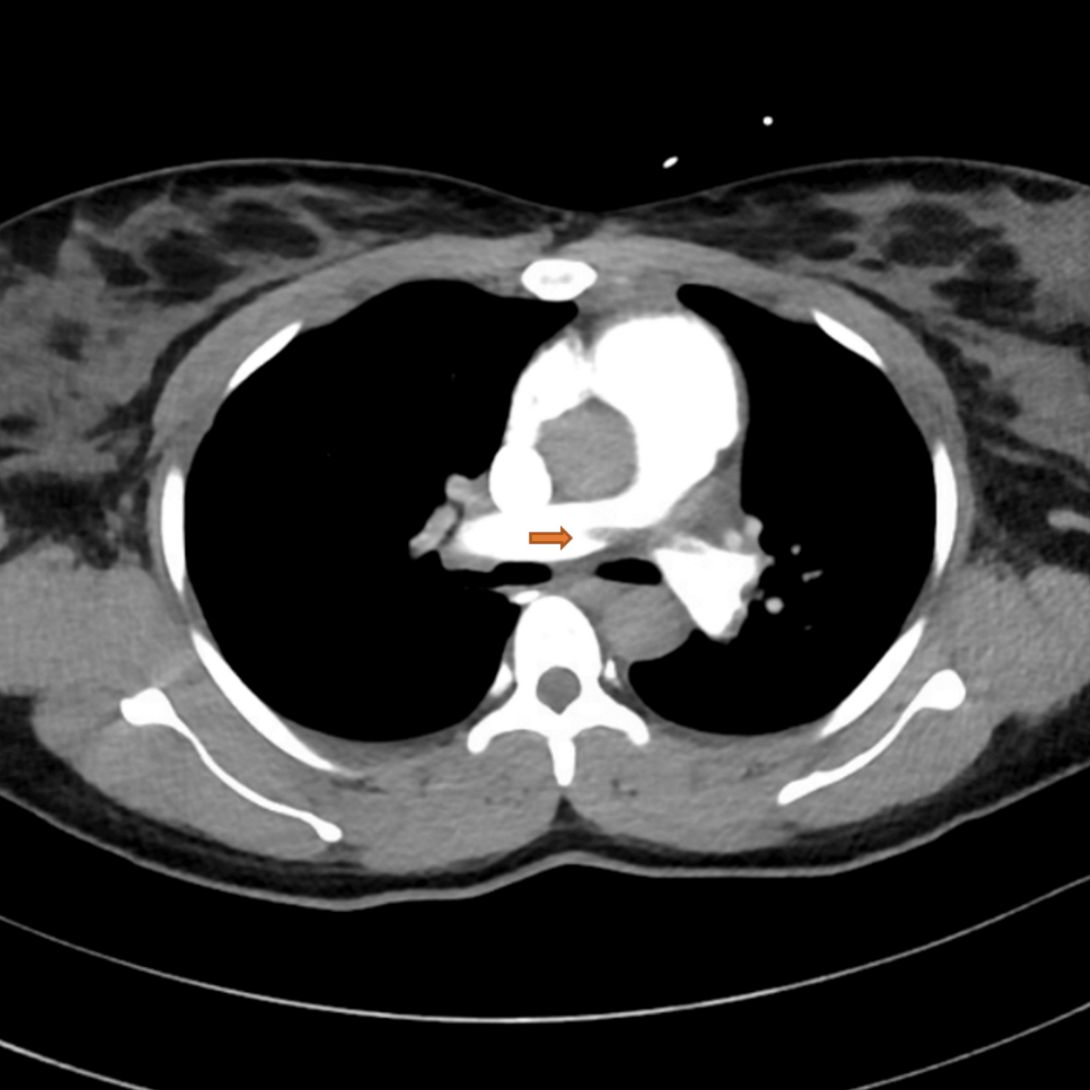
Computed tomography pulmonary angiography (CTPA) showing the extension of the saddle embolus at the pulmonary trunk bifurcation.

**Figure 4:**
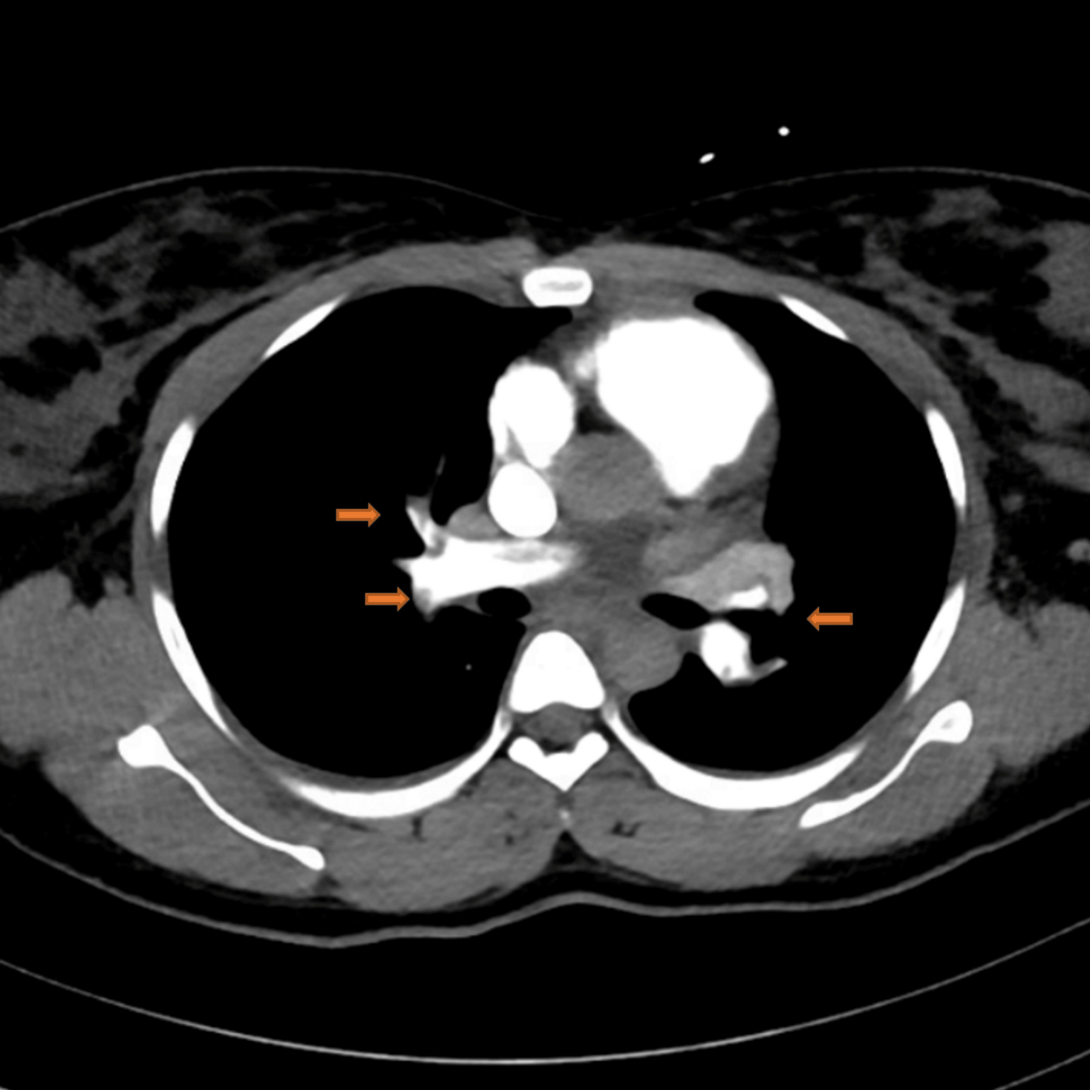
Computed tomography pulmonary angiography (CTPA) showing the extension of the saddle embolus with almost total occlusion of bilateral lobar pulmonary arteries.

**Figure 5:**
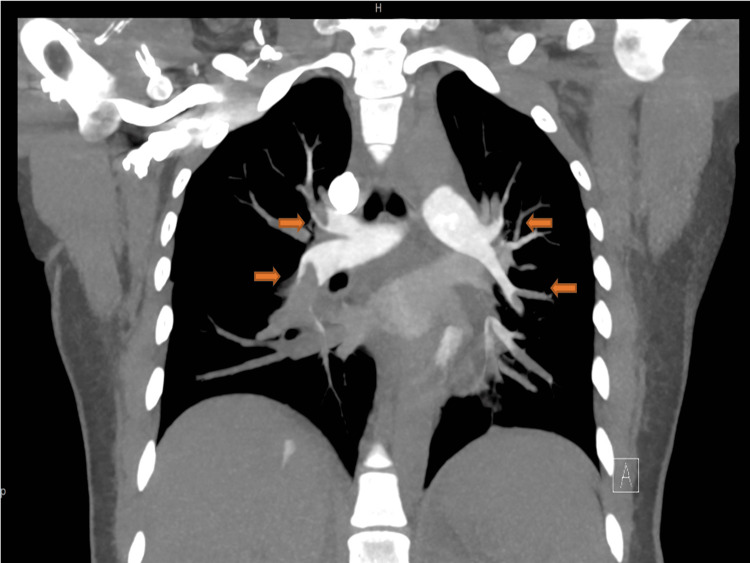
Computed tomography pulmonary angiography (CTPA) showing the extension of the saddle embolus with almost total occlusion of bilateral lobar pulmonary arteries.

A pulmonology consultation was obtained, and a CT pulmonary angiography (CTPA) with abdominal shielding demonstrated extensive bilateral pulmonary emboli, including a saddle embolus, lobar artery occlusions, and segmental/subsegmental involvement, with a small right pleural effusion (Figures 2-5). 

Interventions

The patient was transferred to the intensive care unit (ICU), and thrombolytic therapy was kept on standby. After a multidisciplinary discussion involving pulmonology and interventional radiology, a mechanical thrombectomy was planned. In preparation for the high-risk procedure, the patient was intubated, and central venous and arterial lines were inserted. A heparin bolus followed by infusion was initiated prior to the intervention.

During catheter advancement via the right central venous access into the pulmonary artery, the patient developed cardiac arrest. Cardiopulmonary resuscitation (CPR) was carried out as per the Advanced Cardiovascular Life Support (ACLS) protocol [10], and the catheter was withdrawn to the inferior vena cava. IV alteplase 50 mg was administered over 2 minutes. Return of spontaneous circulation (ROSC) was achieved after two cycles; a second cardiac arrest occurred, followed by sustained ROSC. The procedure was aborted, and the catheter was left in place temporarily due to bleeding risk. An additional alteplase infusion (50 mg over one hour) was administered.

ICU course

The patient developed minimal vaginal bleeding and blood-stained endotracheal secretions. Lower limb doppler ultrasound demonstrated partial venous thrombosis involving the left tibial vein and right femoral vein. Repeat TTE revealed a mildly dilated right ventricle with free wall hypokinesia and an echogenic right atrial mass, consistent with thrombus.

Due to persistent blood-stained endotracheal secretions, an otolaryngology consultation was obtained, and a throat pack was placed with a plan for reassessment after three days. The right femoral catheter was removed, and hemostasis was achieved with manual compression. Transvaginal ultrasonography showed a thickened uterine wall without a visible gestational sac and minimal pelvic free fluid; gynecology assessed this as a missed abortion, requiring no active intervention.

The patient subsequently coughed up the throat pack, prompting otolaryngology re-evaluation. Bedside glidescope examination revealed no mucosal injury, with only clotted supraglottic secretions, which were managed conservatively. She was maintained on intravenous ceftriaxone during her ICU stay. As per cardiology recommendations, heparin infusion was continued for management of the right atrial thrombus. The patient remained sedated and mechanically ventilated with close hemodynamic monitoring.

On day 4 of admission, the patient was successfully extubated and transitioned to high-flow nasal cannula (HFNC), maintaining hemodynamic stability and improved oxygenation without respiratory distress. By day 5, she was comfortable on room air with significant clinical improvement. Troponin-I and β-hCG levels demonstrated a downward trend, and invasive lines were removed. A repeat TTE showed a smaller echogenic mass (1.3 × 1.2 cm) attached to the interatrial septum, with normalization of right atrial and ventricular size. Cardiology advised continuation of heparin infusion and further evaluation with transesophageal echocardiography (TEE).

As her condition stabilized, she was transferred from the ICU to the medical ward. Anticoagulation was transitioned from heparin to therapeutic enoxaparin and subsequently to a novel oral anticoagulant (NOAC) (rivaroxaban), with a planned duration of six months. TEE confirmed a right atrial thrombus measuring 1.5 × 0.9 cm. Given her stable condition, no further intervention was required, and the current management was continued.

Outcome

After a 10-day hospital course, the patient showed steady recovery. She was discharged home in stable condition on Rivaroxaban, and follow-up was arranged with Internal Medicine, Cardiology, and Pulmonology for continued care.

## Discussion

Venous thromboembolism (VTE), encompassing deep vein thrombosis (DVT) and PE, is a leading cause of maternal morbidity and mortality, occurring in approximately one in 1,000-2,000 pregnancies and conferring a five- to 10-fold increased risk compared with non-pregnant women of similar age [1,2]. Pregnancy induces a physiological hypercoagulable state fulfilling Virchow’s triad through progesterone-mediated venous stasis, endothelial activation related to placentation, and increased procoagulant factors with reduced fibrinolytic activity [3, 5]. Established maternal risk factors include prior VTE, age >35 years, obesity, multiparity, medical comorbidities, pre-eclampsia, and immobility [6, 7]. Although thromboembolic risk persists throughout gestation, the postpartum period, particularly the first six weeks, carries the highest vulnerability [6].

Syncope and seizures are uncommon but clinically significant manifestations of acute PE. Syncope has been reported in approximately 13% of PE cases and is typically associated with massive or high-risk embolism, where abrupt pulmonary vascular obstruction results in acute right ventricular (RV) failure, reduced left ventricular preload, and sudden cerebral hypoperfusion [4]. Acute RV pressure overload may also precipitate malignant arrhythmias or conduction disturbances, further contributing to transient loss of consciousness. Seizure-like activity is rare (<1%) and is thought to result from severe hypoxemia and global cerebral hypoperfusion rather than primary epileptogenic pathology [4]. Pregnancy may further lower the threshold for such presentations due to increased oxygen consumption, reduced functional residual capacity, and limited cardiopulmonary reserve, thereby amplifying the hemodynamic consequences of acute PE [1, 5, 7].

Diagnosis in this case was particularly challenging because early pregnancy both increased thromboembolic risk and introduced competing diagnostic considerations, including ectopic pregnancy and implantation bleeding. Persistent tachycardia, hypoxemia, metabolic acidosis, ECG findings (sinus tachycardia and S1Q3T3), and RV dilatation on bedside TTE ultimately redirected suspicion toward PE, later confirmed by CT pulmonary angiography (CTPA). Current international guidance supports prompt imaging when clinical suspicion for PE is high, even during pregnancy, as delayed diagnosis poses greater maternal risk than fetal radiation exposure when appropriate shielding is used [3,6].

Physiological elevations in D-dimer during pregnancy reduce its standalone diagnostic utility, necessitating pregnancy-adapted diagnostic strategies. The Pregnancy-adapted YEARS (ARTEMIS) algorithm is a diagnostic pathway designed to safely rule out PE in pregnant women while minimizing unnecessary exposure to ionizing radiation from CTPA. It integrates clinical assessment with adjusted D-dimer thresholds and remains the most validated approach, demonstrating a low failure rate (0.21%) while safely reducing the need for CTPA [8]. The pregnancy-adapted YEARS (ARTEMIS) algorithm is a freely available, non-proprietary clinical decision tool and does not require licensing for clinical or academic use [8]. This strategy is increasingly supported by European and international consensus statements as a pragmatic, radiation-sparing diagnostic pathway in pregnancy.

A bedside cardiac point-of-care ultrasound (POCUS) plays a crucial adjunctive role in suspected high-risk PE, particularly in hemodynamically unstable patients. While its negative predictive value is limited (~50%), specific echocardiographic findings such as McConnell's sign and reduced Tricuspid Annular Plane Systolic Excursion (TAPSE) are 100% specific, while RV dilatation and a D-shaped left ventricle indicate RV pressure overload with ~80% sensitivity [11]. In this case, a quick FAST was performed to look for hemoperitoneum or hemopericardium, but a bedside TTE performed later was pivotal in escalating care and prioritizing definitive imaging, highlighting the significance of a bedside cardiac POCUS.

Management of PE in pregnancy requires coordinated multidisciplinary care involving emergency medicine, obstetrics, cardiology, pulmonology, neurology, and critical care. Low-molecular-weight heparin (LMWH) remains the anticoagulant of choice during pregnancy due to its safety profile and lack of placental transfer [3]. In cases of massive or hemodynamically unstable PE, systemic thrombolysis or catheter-directed interventions may be lifesaving, although pregnancy-specific evidence remains limited. Emerging data suggest that mechanical thrombectomy may improve RV function and hemodynamics in selected high-risk patients, supporting careful consideration when maternal survival is threatened [12].

Early recognition, prompt imaging, and decisive multidisciplinary intervention were central to the favorable outcome in this case, despite an atypical neurological presentation and the complexities introduced by early pregnancy.

No proprietary scoring systems or licensed clinical tools were used in this case report.

## Conclusions

This case highlights an atypical presentation of massive PE in early pregnancy, initially manifesting as syncope and seizure. It underscores that PE should remain a key consideration in pregnant patients with sudden loss of consciousness or convulsive activity, particularly when accompanied by tachycardia, hypoxemia, or elevated lactate. Prompt diagnosis and maternal stabilization must take priority over minimal fetal radiation concerns. A high index of suspicion supported by a multidisciplinary and algorithm-driven approach is essential for optimizing outcomes.

Future research should focus on refining trimester-specific D-dimer thresholds, validating pregnancy-adapted YEARS/ARTEMIS algorithms across populations, and clarifying the role of point-of-care echocardiography and non-ionizing imaging. A deeper understanding of the pathophysiology behind atypical neurological PE presentations may further improve early recognition and guide tailored management in this vulnerable patient population.
